# Query for the Readers of the Homœopathic Physician

**Published:** 1890-03

**Authors:** John Hall

**Affiliations:** Victoria, British Columbia


### QUERY FOR THE READERS OF THE HOMŒOPATHIC PHYSICIAN.

In case a person is under homœopathic treatment for chronic maladies and he takes an acute form of disease—such as "la grippe"—can he give a lower potency for the recent affection, say 2C, and how far may such treatment be proceeded with? Will the 2C allow the higher one to go on at the same time?

John Hall, Sr., M. D.

Victoria, British Columbia.

